# Plasticity and seasonality of the vertical migration behaviour of Antarctic krill using acoustic data from fishing vessels

**DOI:** 10.1098/rsos.230520

**Published:** 2023-09-27

**Authors:** Dominik Bahlburg, Lukas Hüppe, Thomas Böhrer, Sally E. Thorpe, Eugene J. Murphy, Uta Berger, Bettina Meyer

**Affiliations:** ^1^ Forstliche Biometrie und Systemanalyse, Technische Universität Dresden, Pienner Straße 8, 01737 Tharandt, Dresden, Germany; ^2^ Helmholtz Centre for Environmental Research Leipzig, Permoserstraße 15, 04318 Leipzig, Germany; ^3^ Neurobiology and Genetics, Julius-Maximilian-Universität Würzburg, Am Hubland, 97074 Würzburg, Germany; ^4^ Alfred-Wegener-Institute for Polar and Marine Research, Am Handelshafen 12, 27570 Bremerhaven, Germany; ^5^ Friedrich-Alexander-Universität Erlangen-Nürnberg, Schloßplatz 4, 91054 Erlangen, Germany; ^6^ Ecosystems, British Antarctic Survey, High Cross, Madingley Road, Cambridge CB3 0ET, UK; ^7^ Institute for Chemistry and Biology of the Marine Environment, Carl von Ossietzky University Oldenburg, Carl-von-Ossietzky-Straße 9-11, 26111 Oldenburg, Germany; ^8^ Helmholtz Institute for Functional Marine Biodiversity, Ammerländer Heerstraße 231, 26129 Oldenburg, Germany

**Keywords:** Antarctic krill, vertical migration behaviour, seasonal vertical migration

## Abstract

Understanding the vertical migration behaviour of Antarctic krill is important for understanding spatial distribution, ecophysiology, trophic interactions and carbon fluxes of this Southern Ocean key species. In this study, we analysed an eight-month continuous dataset recorded with an ES80 echosounder on board a commercial krill fishing vessel in the southwest Atlantic sector of the Southern Ocean. Our analysis supports the existing hypothesis that krill swarms migrate into deeper waters during winter but also reveals a high degree of variability in vertical migration behaviour within seasons, even at small spatial scales. During summer, we found that behaviour associated with prolonged surface presence primarily occurred at low surface chlorophyll a concentrations whereas multiple ascent–descent cycles per day occurred when surface chlorophyll a concentrations were elevated. The high plasticity, with some krill swarms behaving differently in the same location at the same time, suggests that krill behaviour is not a purely environmentally driven process. Differences in life stage, physiology and type of predator are likely other important drivers. Finally, our study demonstrates new ways of using data from krill fishing vessels, and with the routine collection of additional information in potential future projects, they have great potential to significantly advance our understanding of krill ecology.

## 1. Introduction

Antarctic krill (*Euphausia superba*, hereafter krill) is a highly abundant crustacean of disproportionate importance to the functions of the Southern Ocean ecosystem [1–3]. Not only is krill a key component of the pelagic food web, it is also the target of a growing commercial fishery [4]. The krill fishery typically operates most of the year, excluding the late winter/early spring months of September and October, with the main fishing grounds being located in the Atlantic sector of the Southern Ocean.

The Commission for the Conservation of Antarctic Marine Living Resources (CCAMLR) was formed in 1982 and is responsible for managing this fishery. More recently, in the face of increasing krill catches, the resurgence of some species of large krill predators [5,6] and the increasing impacts of climate change on key krill habitats [7–9], calls have been made to develop a more adaptive and sustainable management strategy that protects the integrity of the Southern Ocean ecosystem and its functions [4,10–14]. Although highly desirable, such a management plan requires solid knowledge of the ecology of krill, its population dynamics and temporospatial distribution. Many of these aspects of krill ecology are still poorly resolved, and in this study we aim to improve our understanding of krill vertical migration behaviour and its underlying mechanisms.

Diel and seasonal vertical migration are an integral part of zooplankton, and especially krill, ecology [15–17] and of great importance for biogeochemical cycles [18,19], species interactions, and spatial distribution through advection by depth-varying ocean currents [20]. Investigating vertical migration behaviour also yields information about the physiological functioning of the migrating organisms [21,22] and contributes to our overall understanding of the marine pelagic ecosystem. Observations over many years have shown that krill swarms can ascend and descend in the water column in synchrony with the daily light cycle [23–25]. This diel vertical migration (DVM) is thought to be a behavioural adaptation to the increased mortality risk from visual predators during the day and the urge to feed in the productive surface layers [26,27]. As a consequence, krill swarms have been observed to move to the surface after sunset to feed, and descend into deeper waters during the day to avoid predation [23–25]. While DVM is commonly seen in krill behavioural data, it is not ubiquitous and a variety of behaviours from reverse DVM to no vertical migration have been observed and reported [23,28–31]. The mechanisms explaining this variability are not clear but it has been suggested that reverse DVM may be caused by the presence of planktivorous fish [23,32], and that krill swarms remain near-surface in their high-latitude habitats during summer when illumination differences between day and night are less pronounced or even absent [29]. Less is known about the seasonal vertical migration (SVM) of krill. SVM describes seasonal shifts in mean residence depth and has predominantly been observed in polar zooplankton taxa [17,33,34]. Organisms performing SVM typically spend the winter months at increased depths while being shallower in summer [15,33,34]. SVM by Antarctic krill has not been fully determined with some observations indicating a shift to deeper waters during winter [35,36] and others reporting the presence of krill close to the surface, especially when sea ice is present [37,38]. These differences suggest that environmental conditions affect the seasonal depth distribution of krill.

Some of these uncertainties arise from the common limitation of many studies of krill vertical migration in that they only provide snapshots of behavioural dynamics over a few days or weeks, with a strong bias towards summer observations [31,39]. In some studies, data from multiple research campaigns were aggregated but these observations were still restricted to the summer months [25] or based on multiple shorter-period observations [40] with winter typically missing. The use of acoustic instruments on moorings permits the study of the vertical distribution of Southern Ocean zooplankton over longer time periods, but these data are limited to one location [33]. It can also be difficult to identify the recorded species as validating net samples do not exist for the duration of the mooring deployment. There is no perfect solution for these issues as research campaigns in the Southern Ocean are expensive, and getting research time on the existing research platforms is competitive. On the other hand, the commercial krill fishing fleet operates almost year-round, targeting krill swarms in a variety of habitats, and hence providing a potential additional source of observations and data. The advantage of data from fishing vessels is that the vessels operate for much longer time periods than individual field campaigns, and that they explicitly target krill swarms across seasons. Although the vessels do not fish in fully ice-covered waters, they still operate in some of the key krill habitats in the Atlantic sector of the Southern Ocean such as the Antarctic Peninsula, the South Orkney Islands, the Scotia Sea and South Georgia [1,41]. Although CCAMLR deploys independent fishery observers on board these vessels, no systematic programme exists to use the vast amount of information that these vessels could provide on krill ecology and behaviour [4].

In this study, we demonstrate the value of data collected by krill fishery vessels to provide detailed information on the seasonal and diel vertical distribution of krill swarms. We analyse eight months of continuous acoustic recordings that were collected with an ES80 echosounder (Kongsberg Maritime AS) from December 2020 to July 2021 on board the FV *Antarctic Endurance*, a commercial krill fishing vessel operated by Aker BioMarine. Two previous studies used data from the Japanese krill fishing fleet to describe dynamics of seasonal vertical krill distribution [35,36], mainly based on the seasonal variation of net depths and demographic indices of the catch. By using acoustic data, we are able to provide detailed analyses of krill swarm vertical distribution over the upper 250 m of the water column in space and time. Although krill are known to occur at much greater depths [42–45], the main vertical distribution of krill swarms is thought to be in the upper 300 m. In a review of 30 studies assessing the vertical distribution of krill swarms during summer, Schmidt *et al.* [44] found that typically 80–98% of the krill biomass is found in the upper 200 m of the water column. We analysed the vertical migration behaviour of krill from qualitative and quantitative perspectives and considered the observed behavioural variability in relation to environmental conditions. The study demonstrates ways in which krill fishing vessels could contribute to a better understanding of krill ecology and consequently, improved management strategies. We conclude by providing recommendations for additional data that could be collected with relatively little effort on board fishing vessels to allow for even more detailed analyses.

## 2. Methods

Our dataset is based on eight consecutive months of acoustic backscattering data recorded from the krill fishing vessel FV *Antarctic Endurance* (December 2020–July 2021). Raw acoustic data were not available for the investigated time period. Instead, we developed a novel method to reconstruct the backscattering signal from a dataset of greater than 18 000 screenshots that displayed the visualized signal received by the 200 kHz band of the echosounder (documented in Bahlburg *et al.* [46]). The method matches the RGB-values of each screenshot pixel with the RGB-values of the colour scale used for data visualization. Based on the closest matching colour using Euclidean distance of the RGB-values, and its relative position on the colour scale, a signal strength is assigned to each pixel to create a dataset that can be processed in similar ways to raw acoustic data. Importantly, due to the loss of some information when using screenshots (e.g. transducer angle, water temperature and other associated data normally recorded by the ES80), this dataset is not suitable for quantifying krill biomass. However, the data still capture the shape and location of krill swarms, allowing for detailed analyses of the dynamics of vertical krill distribution. For the majority of the investigated time period, data were available for the upper 250 m of the water column. On a few occasions, only the upper 150 m were displayed in the screenshots. In these cases, the FV *Antarctic Endurance* was fishing at very shallow depths, suggesting that the krill swarms were also very shallow and, therefore, we assume that no information about their vertical position was lost. This does, however, mean that no information on krill swarms beneath this depth was captured. We confirmed the validity of the screenshot processing approach by comparing the derived dataset with raw data for shorter periods where raw data were available. In general, the reconstructed behavioural patterns and krill biomass distribution metrics, such as Centre of Mass, were in close agreement between the screenshot-based and raw acoustic data [46].

The robustness of our analyses largely depends on the assumption that the isolated backscattering signal represents Antarctic krill and not other taxa. We minimized this risk by restricting our analyses to time periods where the FV *Antarctic Endurance* was actively fishing and therefore targeting krill swarms. With bycatch ratios of 0.1–0.3% [47], the krill fishery is almost monospecific, and fishing operations would have stopped if non-target species were being caught in significant proportions at any time. Furthermore, the net depth was visible in the raw screenshots and was used to confirm that the analysed backscattering signals did indeed represent krill. Some of the authors of this study spent a total of greater than eight months aboard the FV *Antarctic Endurance* over the course of three fishing seasons (2019/2020, 2020/2021, 2021/2022) and our observations verify that the catch consists almost exclusively of *Euphausia superba*. The high echosounder frequency of 200 kHz also interacts more strongly with smaller organisms, giving zooplankton such as krill comparative dominance in the recorded backscattering signal over fish, cephalopods or other taxa. During fishing operations, the vessel moved at a speed of 1.5–2 knots.

### 2.1. Cruise track

**Figure 1.**
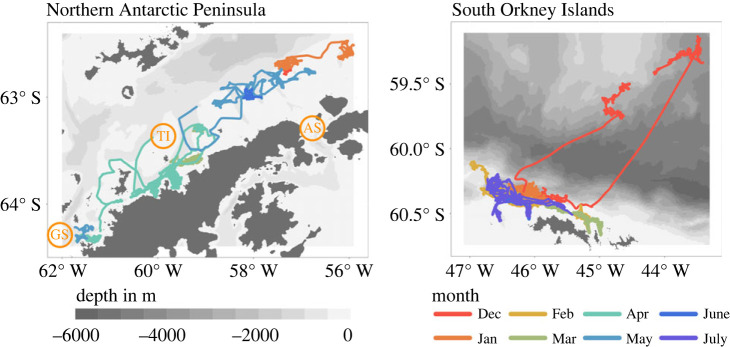
Position of the FV *Antarctic Endurance* when actively fishing from December 2020 to July 2021 (coloured by month). Orange labels in left panel: TI, Tower Island; GS, Gerlache Strait; AS, Antarctic Sound.

The FV *Antarctic Endurance* started its 2020/2021 fishing season at the South Orkney Islands in December 2020 (figure 1). It remained there, with the exception of a short trip to the Bransfield Strait in January, until March before moving south to the Gerlache Strait region. Following approximately six weeks of fishing operations in its southernmost fishing grounds, the FV *Antarctic Endurance* moved back north into the Bransfield Strait where it fished throughout May until early June. After a brief period of fishing at the South Orkney Islands in early June, the FV *Antarctic Endurance* continued to South Georgia for offloading operations and returned to fish the South Orkney Islands shelf break in late June and July.

### 2.2. Quantifying krill behaviour

**Figure 2.**
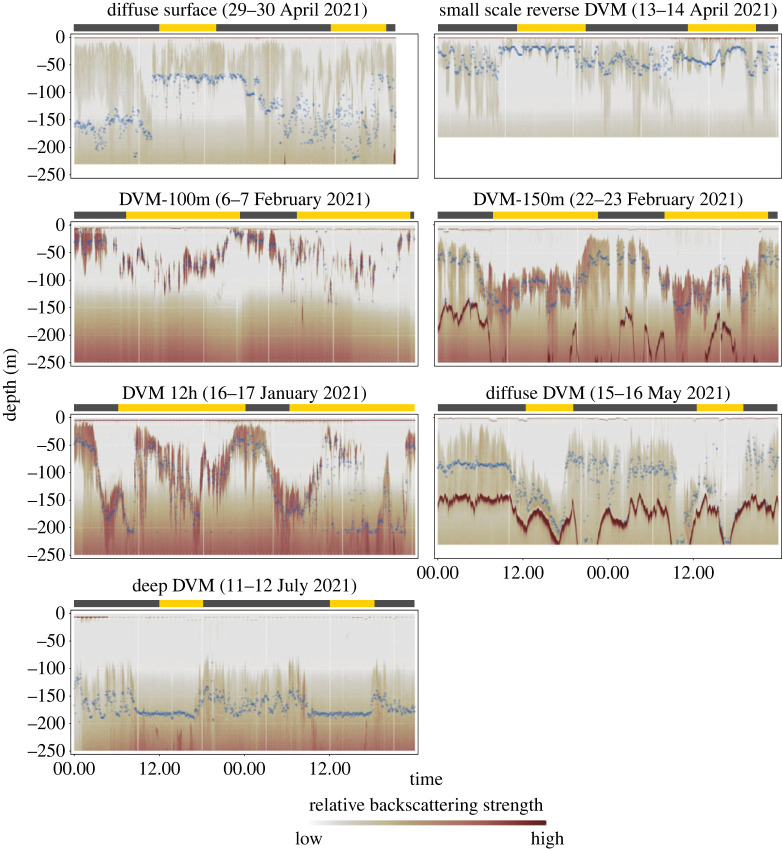
Classification of various behaviours exhibited by krill swarms from December 2020 to August 2021. The behavioural classes are mainly based on swarm characteristics (dispersed, compact) and the way their vertical position was synchronized with the daily light cycle. The blue dots represent the location of the Centre of Mass describing the central depth of krill biomass. The Centre of Mass was a robust metric for most behaviours, but could occasionally give misleading results, such as for ‘diffuse surface’ behaviour shown here. The very high relative backscattering strength showing up in some panels in depth greater than 150 m, e.g. in the ‘diffuse DVM’ panel, represents the sea floor whereas the diffuse signal that appears at approximately 100 m and increases with depth represents instrument noise. The bars at the top of the plots visualize the day (yellow)–night (dark grey) cycle at the given time period.

To investigate seasonal and regional patterns in krill behaviour, we classified the observed behaviour into seven qualitative categories after visually inspecting the dataset (categories shown in figure 2, the full visualized dataset is shown in electronic supplementary material, appendix figures S5–S12). The classification was based on the depth, swarm properties and the synchronization of mean vertical position with the diel light cycle (table 1). We further calculated the Centre of Mass to quantify the average vertical position of krill biomass and analysed its dynamics across regions and seasons. The Centre of Mass [48] was determined after de-noising the dataset according to the algorithm described by De Robertis & Higginbottom [49] and after removing signals from the sea floor and below (the full processing procedure for isolating the biomass signal is described in [46]). The Centre of Mass represented the average depth of biomass reasonably well for most behaviours (electronic supplementary material, appendix figures S5–S12). However, its accuracy decreased when the swarms were either very dispersed, or when the overall backscattering strength was low (e.g. at ‘diffuse surface’ behaviour in figure 2). In addition, the de-noising of the data and hence the calculation of the Centre of Mass was restricted to the upper approximately 200 m due to the strong increase in instrument noise at greater depths (see ‘deep DVM’ in figure 2). For these reasons, we used the Centre of Mass only for a broad seasonal comparison of vertical biomass distribution, which was relatively insensitive to these inaccuracies, and not for more detailed analyses.

**Table 1.** Classification criteria for vertical migration behavioural classes.

behaviour class	characteristics
diffuse surface	swarms are dispersed from the surface to 100 m; vertical swarm position not strongly synchronized with daytime
small scale reverse DVM	swarms change vertical position according to daytime; night residence depth deeper than day residence depth
DVM-100m	swarms change vertical position according to daytime; night residence depth near surface, day residence depth around 100 m
DVM-150m	swarms change vertical position according to daytime; night residence depth near surface, day residence depth around 150 m or deeper
DVM 12h	DVM as in ‘DVM-150m’ but secondary and tertiary ascents to the surface visible during the day
diffuse DVM	swarms change vertical position according to daytime; swarms are deep and contracted during the day, and dispersed throughout the water column during the night
deep DVM	swarms change vertical position according to daytime; night residence depth around 150 m, day residence depth around greater than 250 m

### 2.3. Data analysis

We analysed the behavioural data in relation to ambient environmental conditions comprising of photoperiod, satellite-derived surface chlorophyll a concentrations (as a proxy for food availability), sea surface temperature, sea floor depth and ocean velocities. Photoperiod, defined as the period between local sunrise and sunset, was calculated using the R package *suncalc* [50] for each time point at the respective vessel position. Daily surface chlorophyll a data were retrieved from L4 processed satellite observations available at the Copernicus Marine Service (4 km × 4 km resolution, doi:10.48670/moi-00281). Chlorophyll a data were only available until 25 April 2021 due to lack of sunlight in austral winter which prevents the measurement of ocean surface spectral properties. Bathymetric data were extracted from the International Bathymetric Chart of the Southern Ocean (500 m × 500 m resolution [51]), daily sea surface temperature and ocean velocity data (0–380 m, 30 depth levels) were extracted from the Operational Mercator global ocean analysis accessible at the Copernicus Marine Service (0.083° × 0.083° resolution, https://doi.org/10.48670/moi-00016). We then extracted the environmental information (depth, sea surface temperature, surface chlorophyll a) along the cruise track at the corresponding time points and aggregated them for the box plots shown in figure 5. There was no information available on the body length of the krill or any other physiological or demographic information. Distance to coast was calculated using the *gDistance* function from the *rgeos* package [52], which determines the Cartesian minimum distance between two spatial objects (in our case vessel position and coastline).

Data handling, analysis and visualization were carried out in the R programming language [53] using the packages *tidyverse* [54], *terra* [55], *tidyterra* [56] and *scico* [57]. Shapefiles of the Antarctic coastlines and islands shown in the maps were taken from the SCAR Antarctic Digital Database [58].

## 3. Results

### 3.1. Spatio-temporal dynamics of krill behaviour

**Figure 3.**
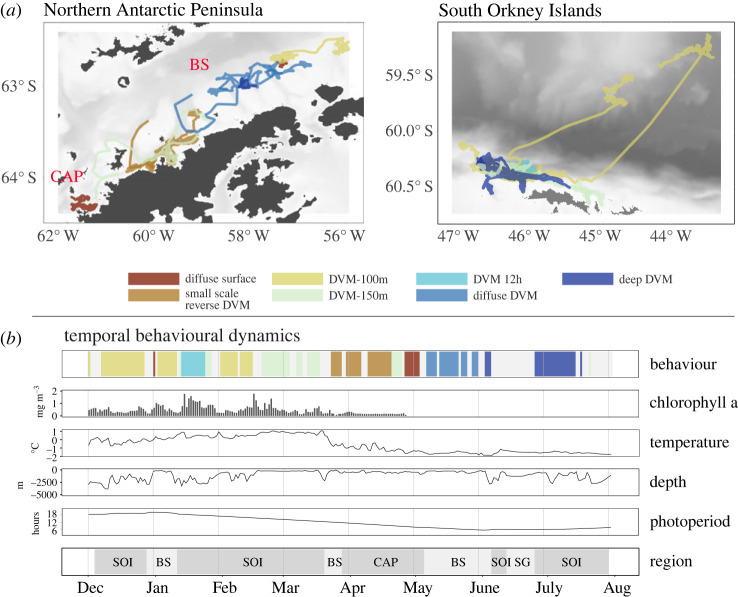
(*a*) Cruise track of the FV *Antarctic Endurance* coloured according to the observed krill behaviour in a given location. In (*b*), the temporal dynamics of behaviour, surface chlorophyll a concentration (limited to 1 December 2020–25 April 2021), sea surface temperature, depth and region are shown. Grey areas in the behaviour timeline represent periods when the *Antarctic Endurance* was moving between fishing grounds or when krill swarms were visible for much less than 24 h, allowing no characterization of vertical migration behaviour. No data were available for South Georgia. Abbreviations for regions are: SOI, South Orkney Islands; BS, Bransfield Strait; CAP, central Antarctic Peninsula (areas south of Bransfield Strait); SG, South Georgia.

The period from December (mid-summer) to mid-March (early autumn) was characterized by three distinct behaviours (figure 3). At the beginning of December (mid-summer), when the fishery vessel was at the shelf slope of the South Orkney Islands with depths of 300 → 2000 m and surface chlorophyll a concentration of 0.3–0.5 mg m^−3^, mainly ‘DVM-100m’ and occasionally ‘diffuse surface’ behaviour was observed meaning that significant proportions of the krill swarms spent substantial amounts of time close to the surface (in the upper 50 m), irrespective of time of day. This was also the case during a brief trip to the Bransfield Strait in late December/early January (summer) where krill swarms showed ‘diffuse surface’ behaviour under surface chlorophyll a concentrations approximately 0.8 mg m^−3^ (figure 3; electronic supplementary material, appendix figures S5 and S6). Such signals could be caused either by a swarm dispersing through the water column, or by many individuals ascending and descending asynchronously. A sharp shift in observed behaviour occurred in mid-January when the FV *Antarctic Endurance* returned to the shelf slope at the South Orkney Islands (slightly more west than in December) where it encountered krill swarms exhibiting ‘DVM 12h’ behaviour (alternating between approx. 50 and 180 m), often including a distinctive secondary ascent at noon. More specifically, all encountered krill swarms made an ascent to the surface after sunset (24 h period) and some performed a secondary (following a 12 h period) or tertiary ascent during the day (electronic supplementary material, appendix figure S6). The local environment was characterized by comparatively deep waters at the shelf break (approx. 2000 m), increased surface chlorophyll a concentrations of greater than 1 mg m^−3^ and surface water temperatures around 0°C (figure 3). The vessel then moved along the shelf slope into shallower waters (400–1000 m) with reduced surface chlorophyll a concentrations (less than 0.5 mg m^−3^), accompanied by a return to ‘DVM-100m’ behaviour (figure 3; electronic supplementary material, appendix figure S7). Another switch in behaviour to ‘DVM-150m’ with an amplitude of 150 m occurred when the vessel moved further onto the shelf (approx. 250 m) in late February (late summer), where surface chlorophyll a concentrations were initially higher than in the previously fished region (0.5–1.5 mg m^−3^; figure 3; electronic supplementary material, appendix figure S8). However, chlorophyll a concentrations in this region decreased over time with no change in observed behaviour. In summary, three very different behaviours characterized by either small or large diel changes in mean swarm depth were observed at local scales in the South Orkney Islands and Bransfield Strait from December (mid-summer) to mid-March (early autumn). While the ‘diffuse surface’ behaviour was associated with shallow waters and chlorophyll a concentrations of less than 0.5 mg m^−3^, ‘DVM-150m’ and ‘DVM 12h’ behaviour occurred in both shallow and deep waters, but was typically associated with elevated surface chlorophyll a concentrations greater than 1 mg m^−3^.

In late March (autumn), the FV *Antarctic Endurance* moved south towards the Bransfield Strait. Similar to the previous visit in January (summer), surface chlorophyll a concentrations were less than 0.5 mg m^−3^, and krill swarms again exhibited ‘diffuse surface’ behaviour (electronic supplementary material, appendix figure S9). After fishing along the mouths of two underwater canyons in depths of approximately 300 m (figure 3), the vessel continued its operations further south starting around Tower Island and continuing its way into the Gerlache Strait throughout April (autumn). This southward shift was accompanied by a strong decrease of surface water temperatures from approximately 1°C to −1°C and a decrease of the daily photoperiod below 12 h. The observed krill swarms exhibited ‘diffuse surface’ and ‘small scale reverse DVM’ behaviours, both associated with shallow swarm distributions and little change in mean swarm depth over the diel cycle (electronic supplementary material, appendix figure S10). Note that the behaviour that we classified as ‘small scale reverse DVM’ mainly consisted of compact near-surface krill swarms during the day, that dispersed into less dense swarms in the upper 40 m during the night (figure 2 and table 1), therefore representing a behaviour that was characterized by prolonged near-surface presence. Ambient surface chlorophyll a concentrations during this time period were at their lowest across the available time period with concentrations less than 0.2 mg m^−3^.

In early May (late autumn), the vessel moved back north to spend the majority of the month fishing in the Bransfield Strait along the mouths of underwater canyons extending from the tip of the Antarctic Peninsula and the Antarctic Sound. Krill behaviour during austral autumn was characterized by ‘diffuse DVM’ meaning that the swarms alternated between a compact formation close to the seafloor during the day and a dispersed state at night, where the biomass signal spread throughout the entire water column (figure 3; electronic supplementary material, appendix figure S11). At this time of the year, no surface chlorophyll a estimates were available, the photoperiod was approximately 7 h, and fishing mainly took place in relatively shallow waters of less than 300 m (with surface water temperatures less than 0°C). On 4 June 2021, the FV *Antarctic Endurance* left the Bransfield Strait.

After a very short period of fishing in the South Orkney Islands and another 13 days of offloading and exploration around South Georgia, fishing resumed on the northwestern shelf slope of the South Orkney Islands on 26 June 2021, in the middle of the austral winter. During this time, the encountered krill swarms exhibited DVM behaviour with their shallowest distribution at approximately 150 m during the nighttime followed by a descent to greater than 250 m at the time of sunrise. This ‘deep DVM’ behaviour was very different from previous observations, as the ascent and descent depths were greater than 100 m, deeper than the observations from the same region earlier in the season (figures 2 and 3; electronic supplementary material, appendix figure S12). The environment at this time of year was characterized by surface water temperatures below −1.5°C, short days with photoperiods of 6 h, and advancing sea ice from the south (the FV *Antarctic Endurance* was fishing in areas with greater than 50% sea ice cover from mid-July; electronic supplementary material, appendix figure S1).

### 3.2. Seasonal patterns

**Figure 4.**
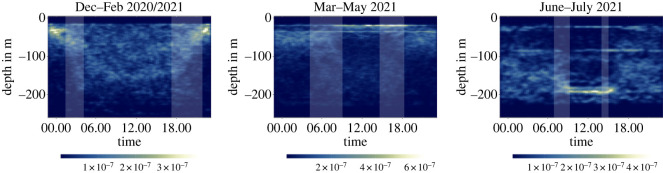
The interplay of vertical distribution and diel light cycles throughout the season. The plots show the two-dimensional kernel density estimation of all Centre of Mass values from a given depth and time of the day in austral summer, autumn and winter (indicative of the relative frequency of occurrence). The light grey bars show the range of sunrise (left bar) and sunset (right bar) times within each season to highlight potential synchronizations of krill behaviour with the diel light cycle. Seasons are defined as follows: summer: Dec–Feb; autumn: Mar–May; winter: June–July. The two horizontal lines at 20 m and 90 m in winter and at 20 m in summer and autumn are artefacts introduced by times when the biomass signal was weak and swarm signal isolation was inaccurate.

To analyse seasonal patterns of vertical krill biomass distribution and behaviour, we grouped the Centre of Mass data into ‘summer’ (Dec–Feb), ‘autumn’ (Mar–May) and ‘winter’ (June–July). It is important to recognize that the Centre of Mass could occasionally give misleading results when the swarms were dispersed, when the backscatter signal was weak, or when the krill swarms were deep. These periods introduced some artefacts that are visible in figure 4. While the high frequency of near-surface Centre of Mass values in March to May 2021 is mainly caused by an extended period from 21 March to 18 April when krill swarms were often shallow (electronic supplementary material, appendix figures S8–S9), it is further amplified by a period from 7 May to 10 May when the krill signal was weak and the Centre of Mass indicated a surface presence, although the swarms were dispersed at 50–150 m (electronic supplementary material, appendix figure S10). Another example is the period from June to July 2021, where the Centre of Mass indicates a daytime residence depth of 200 m, although krill swarms descended to 250 m and below (figures 2 and 4; electronic supplementary material, appendix figure S12). This is caused by high instrument noise with increasing depth, which prevents the De Robertis & Higginbottom de-noising algorithm [49] from isolating biomass signals at depth greater than 200 m for high-frequency bands (200 kHz in our case).

As described previously, the summer months were dominated by four behaviours (‘diffuse surface’, ‘DVM-100m’, ‘DVM-150m’ and ‘DVM 12h’). This is reflected in the Centre of Mass which was almost always shallow (less than 50 m) during the night but could range from 20 to 180 m during the day (figure 4).

During the autumn, the observed vertical migration behaviour changed from behaviours characterized by increased surface presence in April to an alternation of compact near-bottom swarms during the day and dispersed swarms during the night in May. As a result, the Centre of Mass concentrates at depths less than 80 m during the night with three modes of residence depth during the day, one being shallow and the two others being deeper at approximately 120 m and 200 m (figure 4; residence depths greater than 200 m are not captured by the Centre of Mass, see Methods).

In winter, mean krill biomass distribution shifted into deeper waters (residence depths of greater than 100 m at almost all times) with a deep mode of DVM behaviour (figure 4; electronic supplementary material, appendix figure S12). During the day, krill swarms remained in depths greater than 200 m, followed by an ascent to approximately 150 m during the night (electronic supplementary material, appendix figure S12). Therefore, krill still responded strongly to the diel light cycle in winter by migrating up and down the water column.

### 3.3. Environmental covariates and potential drivers of behaviour

Although our data do not allow for a fully mechanistic analysis, we related the different behavioural classes to a suite of environmental variables that have been suggested as drivers of krill behaviour in previous studies: bottom depth (potentially restraining migration depth), distance to coast (serving as a proxy for predation pressure as many air-breathing krill predators hunt in the vicinity of land), photoperiod (photoperiods around 12 h correspond to the strongest diel change in brightness, excluding the effect of clouds), sea surface temperature (water temperature affects metabolic processes) and sea surface chlorophyll a concentration (proxy for feeding conditions near the surface).

**Figure 5.**
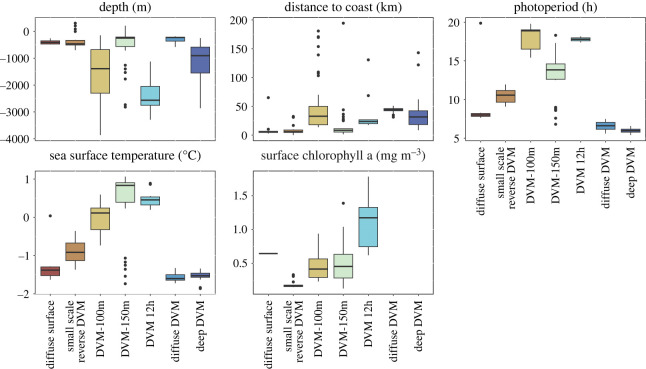
Behavioural classes and associated environmental variables when each behaviour was observed. The boxplots show the median value for each environmental variable (horizontal line), 25th and 75th percentiles (lower/upper hinges of the box) and the whiskers extend to the lowest/highest value or 1.5× the distance between the first and third quartiles from the upper/lower hinges. In the latter case, data outside the range of the whiskers are shown as points. The behavioural classes are ordered along the *x*-axis in an approximate gradient so that from left to right the behavioural classes were increasingly associated with reduced surface presence of krill swarms. Their shading corresponds to figure 3.

In our observations, krill behaviour did not seem to be strongly related to depth of the seafloor (figure 5). For example, qualitatively very different behaviours such as ‘diffuse surface’ presence, ‘DVM-150m’ or ‘diffuse DVM’ could be observed in shallow waters on-shelf. The same applies to distance to coast. Behaviours associated with increased risk of predation due to prolonged periods of near-surface presence (‘diffuse surface’, ‘small scale reverse DVM’) occurred close to shore, and behaviours with clear diel changes in vertical position (‘DVM-100m’, ‘DVM-150m’, ‘DVM 12h’) occurred both close to shore and offshore (figure 5). The photoperiod in our data mainly reflects, on the one hand, the latitudinal movements of the vessel and, on the other hand, the seasonal succession of day length, with the latter being the dominant factor. Overall, the results here correspond to the patterns already shown in figure 4. In summer, when the photoperiod was greater than 15 h, we observed two modes of DVM (‘DVM-100m’, ‘DVM-150m’) and a single day with ‘diffuse surface’ behaviour, which transformed into ‘DVM-100m’. However, the ‘DVM-150m’, which was characterized by very clear diel ascents and descents, was also observed under photoperiods of less than 10 h. ‘Diffuse DVM’ and ‘deep DVM’ occurred in late autumn and winter, when the photoperiod was approximately 5 h. The same is reflected in the association of the different behaviours and sea surface temperature. The patterns here are mainly driven by the seasonal succession of sea surface temperatures, characterized by a cooling in autumn and winter (figure 5). ‘DVM-100m’, ‘DVM-150m’ and ‘DVM 12h’ typically occurred at higher sea surface temperatures in summer, although ‘DVM-150m’ was also observed at temperatures less than −1°C in autumn in the Gerlache Strait. Surface chlorophyll a concentration shows a distinctive pattern where the behaviours characterized by increased surface presence (‘small scale reverse DVM’ and in parts ‘DVM-100m’) tended to be associated with lower surface chlorophyll a concentrations (less than 0.5 mg m^−3^). By contrast, ‘DVM 12h’ was observed when surface chlorophyll a concentrations were greater than 0.5 mg m^−3^. For the ‘diffuse surface’ behaviour, only one chlorophyll a value was available, as later observations of this behaviour were made after 25 April 2021, when no surface chlorophyll a information was available. Similarly, ‘diffuse DVM’ and ‘deep DVM’ behaviour were only observed during winter and hence there are no chlorophyll a data for these behaviours.

**Figure 6.**
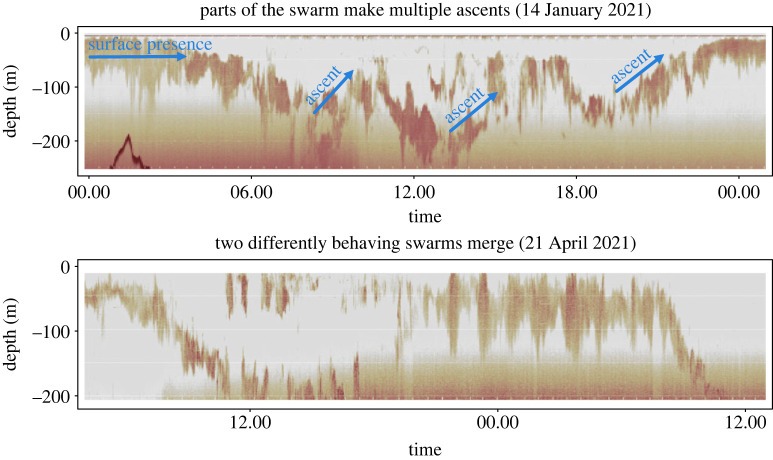
Periods in the dataset where, at a given timepoint and under the same environmental conditions, krill swarms expressed different behaviours. We validated that the different visible backscattering signals represent krill by checking that the FV *Antarctic Endurance* was actively fishing the different swarms (the net depth can be seen in the original screenshot as purple lines, and it was visible that the net depth was alternating between the different biomass signals; electronic supplementary material, appendix figure S2).

Except for the shift of vertical krill swarm position into deeper waters in winter, and the association of ‘DVM-12h’ with high surface chlorophyll a concentrations, there are few other clear patterns in the dataset. Different behaviours can occur in close proximity and under similar environmental conditions (figures 3 and 5), suggesting that krill swarm behaviour is not entirely driven by environmental conditions. In support of this, we found periods when swarms behaved differently under the exact same environmental conditions in one location (figure 6). For instance, in January, we observed krill swarms of which fractions ascended to the surface during the day while others remained at depth (figure 6). In April, in the Gerlache Strait region, the FV *Antarctic Endurance* encountered swarms that were very shallow during the day, while another swarm had just previously descended to 200 m. Eventually, both swarms merged into a single backscattering signal when the deep swarm ascended to the surface around the time of sunset (figure 6). Without additional information, the reasons for these between-swarm differences remain speculative but internal physiological state (hunger) of the individuals within a swarm or body length might play important roles.

## 4. Discussion

The dataset presented in this study provides detailed insights into the variability of krill vertical migration behaviour. Its strengths lie in the amount of information contained in acoustic data and the continuity of the observation of krill swarms over a long period of time, covering three seasons. However, this continuity comes at the expense of local long-term observations since the vessel was constantly moving, tracking krill swarms. This also adds complexity to the data analysis as we cannot be certain when the acoustics show the same swarm, and when new swarms were encountered. However, the same applies to almost all other types of observations, such as those from moorings or monitorings along transects. More importantly, the lack of *in situ* measurements of the physical and biological properties of the water column, as well as length–frequency data characterizing the krill swarms, prevented us from doing more detailed and mechanistic analyses. Diagnostic metrics such as the Centre of Mass or measures of swarm dispersal (not presented here but tested during the analysis) were unfortunately not reliable enough across the different behaviours to conduct additional analyses such as automatically quantifying DVM amplitude. We have therefore kept much of our analysis qualitative and descriptive, with special emphasis of the complexity of the vertical migration behaviour of krill. With these limitations in mind, our results still demonstrate the potential of using fishery acoustics for ecological research on krill, especially considering that much of the missing data in this study could easily be sampled in the future.

### 4.1. Seasonal patterns

The summer was characterized by different modes of DVM (differing in descent depth and the frequency of ascending to the surface) and ‘diffuse surface’ behaviour which was replaced with a more ‘diffuse DVM’ in late autumn and eventually ended in a ‘deep DVM’ in winter. The fact that behaviours associated with increased near-surface presence were more prevalent in the summer months and early autumn is consistent with previous observations [23,28,29]. However, our spatio-temporal data demonstrate how vertical migration behaviour within summer can still be qualitatively very different, even on small scales in the same regions.

A shift of krill swarms into deeper waters in winter has been hypothesized in previous studies [35,36,38,59,60]. However, the underlying data were based on trawl net depths from fishing vessels [35,36], a relatively coarse proxy of swarm depth, mooring data where species identification was not possible [33] or short-term observations [30]. In our continuous data, we could clearly see how the vertical distribution of krill swarms shifted to deeper waters at the same locations in the Bransfield Strait and the South Orkney Islands compared to summer, strongly supporting this hypothesis. Deviations from this winter krill behaviour likely occur in ice-covered waters which represent a very different habitat compared to those sampled in our study. In its high latitude habitat, krill have been observed feeding at very shallow depth under the sea ice in winter, with juveniles and larvae usually being the dominant stages [37,38]. Unfortunately, due to the relatively high noise in the Centre of Mass data presented in this study, analyses beyond the description of coarse seasonal differences in the vertical biomass distribution were not possible.

### 4.2. Diel patterns

**Figure 7.**
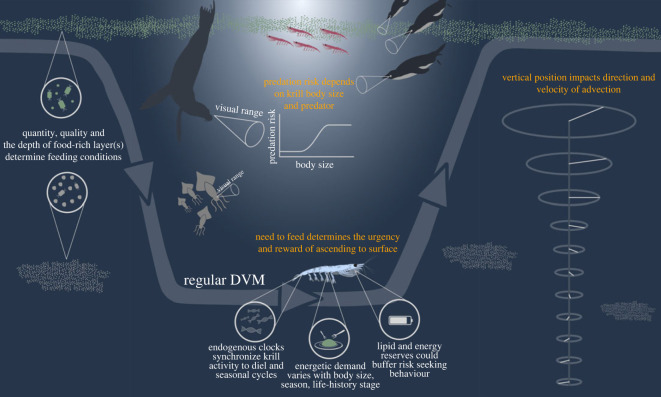
Conceptual figure illustrating important mechanisms and implications that may contribute to the variability in krill behaviour that we observed in our dataset.

Although our dataset does not allow us to confidently identify the drivers of the full complexity of observed behaviours, we can still discuss previously proposed drivers in the context of our data, and hypothesize new ones that might explain some of the remaining variance (figure 7).

If DVM is a trade-off between maximizing food intake and minimizing predation risk, different mechanisms contribute to the weighting of this trade-off: the quantity, quality and vertical location of food sources determine the potential reward of taking the risk to ascend to the surface. It has been recognized in previous studies that feeding conditions can play an important role shaping krill and zooplankton behaviour [15,36,39,40]. In Godlewska [40], chlorophyll a concentration has been named as an important factor driving the amplitude of DVM of krill but the observations that led to these conclusions were gathered from multiple shorter-term studies in different regions of the Southern Ocean. Our data generally support this hypothesis, as DVM with high amplitudes and frequencies was predominantly observed when chlorophyll a concentrations were increased. At the highest surface chlorophyll a concentrations, swarms performed vertical migrations with several ascents and decents per day. The occurrence of multiple ascents and descents agrees well with the observations of Tarling & Johnson [61], who proposed multiple ascent–descent cyles due to a satiation sinking of krill when the stomach is full, followed by active swimming to the surface after digestion is complete. In line with the variability in our data, there are some qualitative differences between our observations and those of Tarling & Johnson [61]. For example, Tarling & Johnson predict up to three ascents during the night whereas we observed multiple ascents during the day. In addition, Tarling & Johnson [61] describe a maximum depth of 43 m by passive sinking while swarms in our data descended to greater than 100 m. Nevertheless, the observations made in our study and Tarling & Johnson may indicate that krill swarms indeed perform vertical migrations with increased frequency when feeding conditions are exceptionally favourable in order to maximize their energy intake.

To our knowledge, no studies exist that explicitly characterize krill vertical migration behaviour outside of winter under poor feeding conditions. In our data, krill swarms seemed to spend more time near-surface when surface chlorophyll a concentrations were relatively low. It is unclear whether krill swarms spent much time at shallow depths under these circumstances because of low surface chlorophyll a concentrations or whether surface chlorophyll a concentrations were low because krill swarms spent so much time feeding in shallow waters. Krill are very efficient feeders and known for their ability to completely deplete phytoplankton blooms [62]. However, if feeding activity was the reason for the low surface chlorophyll a concentrations, we would expect to observe shallow krill when food was abundant, which was not the case. Given the high abundance of visual and air-breathing predators in the coastal waters of the Antarctic Peninsula [5,6,63], it seems risky to spend so much time near the surface, but arguably, when increased energy requirements coincide with poor feeding conditions, krill may not have many alternatives but to remain near the surface to meet their minimum energy requirements (which are also increased in summer [64–66]). Relationships between the occurrence of constant surface presence and distance from the coast, which have been proposed in other studies [28,67], could not be found in our data.

One consequence of spending more time near the surface is an increased spatial displacement by currents where vertical velocity gradients and shear are strong, as is often the case in coastal waters (see electronic supplementary material, appendix figure S4, where we assessed vertical current speed gradients for the regions and time periods in which the FV *Antarctic Endurance* operated). Whether this is a side-effect or an anticipated outcome of staying near the surface, an increased horizontal displacement could help krill reach new regions with improved feeding conditions (figure 7). Complementary, and as demonstrated in advection modelling studies for Palmer Canyon on the Antarctic Peninsula [68,69], DVM can increase local retention, especially within mesoscale subsurface eddies. Therefore, both behaviours (prolonged surface presence and DVM) may also be energy-efficient mechanisms to move or remain in regions where resources are patchily distributed.

In our winter observations, krill swarms also underwent synchronized ascents and descents with daytime depths of greater than 250 m and nighttime depths of approximately 150 m. Such ‘deep mode’ of DVM has been previously reported [29,30] and, as DVM is usually considered as the adaptation to the trade-off of maximizing food intake while minimizing predation risk [26], it likely represents active foraging behaviour. If true, it raises two questions: what were the krill feeding on at depths of approximately 150 m?; and was there an increased predation risk that forced krill swarms to descend to deeper waters during the day? Although we do not have measurements of water column properties from the time the FV *Antarctic Endurance* was operating at the South Orkney Islands during winter, the ascent depth of approximately 150 m roughly corresponds with the winter mixed layer depth reported in this region [70]. This may indicate that the krill were actively feeding near the pycnocline, which could represent a relatively food-rich layer compared to the unproductive surface waters due to particle accumulation along the sharp water density gradient. In response to the second question, increased predation pressure during the day in winter became evident in the acoustic data, which showed high abundances of air-breathing predators feeding on the krill swarms (electronic supplementary material, appendix figure S3). During these periods, the predator activity was highly synchronized with the krill ascent and peaked during the night, highlighting the behavioural interplay of krill and its predators [71–75].

On a side note, the synchronized ascent of krill swarms from depths greater than 300 m during the dark winter months arguably occurred with ambient light levels below their visual perception threshold [76]. This may indicate the involvement of endogenous clocks to synchronize vertical migration behaviour with the diel light cycle [22,59,77–79], as otherwise it would remain unclear how swarms would be able to time their vertical movements without visual cues.

Finally, the importance and interpretation of the discussed drivers of vertical migration behaviour of krill may be context-dependent, which may be a major cause of the high level of variability we find in the dataset (figure 7). For instance, changing cloud cover influences the underwater light field and, consequently, the meaning of the photoperiod as we considered it in our analysis. Additionally, the question of what constitutes favourable feeding conditions, and therefore the potential reward of ascending to the surface, depends on the current energetic demand of the krill (e.g. the high demand of fatty acids for adult female during embryo production [80–82]), the taxonomic and nutritional composition of the food source [83–85], as well as the season. As a consequence, surface chlorophyll a concentrations of e.g. 0.5 mg m^−3^ may be meaningless when the primary producers are an unsuitable food item for krill. Where the primary producers are suitable prey for krill, a chlorophyll a concentration of 0.5 mg m^−3^ could represent a moderate feeding opportunity in peak summer, but a productivity hotspot in late autumn. The energetic demands, and therefore the potential necessity of krill to adopt risky behaviours, may depend on the life stage, season and recent history of starvation. Finally, predation risk can depend on the type of predator as well as on the krill body size, and consequently, size-specific krill behaviour has been observed in the field [36,40], and confirmed by modelling studies to provide energetic and eco-evolutionary benefits [27,28]. Although we cannot directly account for size-specific krill behaviour in our study, we observed behavioural variability that might indicate such a pattern. In particular, during periods when krill swarms exhibited 12h DVM behaviour, it was visible that the midday secondary ascent was usually performed by a fraction of the swarms, while others remained at depth. A 12 h period of vertical migration behaviour also corresponds well with observations of DVM for juvenile krill [40], a secondary period of diel locomotion activity [77] and with periodic expressions of some genes involved in endogenous clocks [79] although these were not specific to juvenile krill. A mechanistic understanding of this behavioural plasticity and its drivers would significantly advance our understanding of krill ecology and help improve modelling studies that incorporate krill behaviour [28,69,86].

### 4.3. Outlook

The acoustic dataset collected by a commercial krill fishing vessel has enabled analyses of the vertical migration behaviour of krill at a high temporal resolution from austral spring through to early winter, providing new, detailed observations of this important aspect of the ecology of krill. Nevertheless, there are some limitations to our findings: The FV *Antarctic Endurance* cannot fish in fully ice-covered waters, which limits our findings mainly to open water and ice-edge habitats. Krill behavioural patterns in ice-covered waters can differ considerably from those found in our data [37,87]. In addition, it is not known whether our observations are biased towards krill swarms with properties that make them particularly attractive for commercial fishing. The FV *Antarctic Endurance* is a commercial fishing vessel that operates based on economic principles and that arguably targets krill aggregations that allow for maximum yield. However, similar properties of krill swarms are favoured by krill predators [88], which makes our data still highly relevant for understanding the role of krill and its interactions in the Southern Ocean food web. Additional data such as krill length frequency, lipid content, sex and maturity, and associated environmental variables will help further investigate the drivers of krill swarm behaviour in future. Complementary observations from other vessels that simultaneously operated in other regions would further allow for cross-validating our findings and substantially contribute to resolving the mechanisms driving seasonal and regional variability in the vertical migration behaviour of krill.

Our analyses were further limited by the sensitivity of metrics describing the vertical distribution of krill, such as the Centre of Mass. While the Centre of Mass was often robust in describing the mean position of the krill distribution, it could occasionally give misleading results when the distribution was more dispersed or due to data noise, processing artefacts, weak backscattering signals or diving predators interpreted as krill. We tested other metrics that included information on the vertical dispersal of the krill distribution, but these were not useful. More robust processing techniques and new approaches to extracting information and describing the vertical distribution of krill and swarm characteristics are needed to realize the full potential of these data in the future. A potentially important aspect that we were not able to take into account in this study is the effect of artificial light emitted from the ship on the observed krill behaviour, especially on days with short photoperiods in autumn and winter. Recent studies have shown that artificial light can strongly alter the vertical migration behaviour of pelagic organisms [89–91]. Quantifying the magnitude and persistence of this effect is highly important, as it could have strong implications for almost all vessel-based observations in behavioural studies of pelagic organisms conducted to date.

## Data Availability

The entire dataset used for the qualitative analysis of krill behaviour is shown in the electronic supplementary material appendix. Data and relevant code for this research work are stored in GitHub: https://github.com/dbahlburg/krillBehaviour and have been archived within the Zenodo repository: https://doi.org/10.5281/zenodo.8222733 [92]. The data processing prior to analysis is documented at https://www.biorxiv.org/content/10.1101/2023.04.16.537064v1 including a fully reproducible example at https://dbahlburg.github.io/isolateBiomassSignal/. The data are provided in electronic supplementary material [93].
